# Falls and fall-related injuries: prevalence, characteristics, and treatment among participants of the Geelong Osteoporosis Study

**DOI:** 10.3389/fpubh.2024.1454117

**Published:** 2024-10-18

**Authors:** Tewodros Yosef, Julie A. Pasco, Monica C. Tembo, Lana J. Williams, Kara L. Holloway-Kew

**Affiliations:** ^1^Deakin University, Institute for Mental and Physical Health and Clinical Translation (IMPACT), School of Medicine – Barwon Health, Geelong, VIC, Australia; ^2^School of Public Health, College of Medicine and Health Sciences, Mizan-Tepi University, Mizan Teferi, Ethiopia; ^3^Department of Medicine – Western Health, The University of Melbourne, St Albans, VIC, Australia; ^4^Department of Epidemiology and Preventive Medicine, Monash University, Melbourne, VIC, Australia

**Keywords:** falls, fall-related injuries, Geelong Osteoporosis Study, older adults, population based sample

## Abstract

**Background:**

Falls are a significant public health challenge, especially among older adults. In Australia, falls and related injuries incur an annual cost of $2.3 billion. However, there is a scarcity of prevalence data on falls among population-based groups. This study aimed to report the characteristics, circumstances, and treatment for falls and fall-related injuries in a population-based sample of Australian men and women.

**Methods:**

Participants from the Geelong Osteoporosis Study provided cross-sectional data: baseline for men (2001–2006) and 10-year follow-up for women (2004–2008). Falls over the previous 12 months were self-reported by 2,631 participants aged 20–97 years (1,533 men and 1,098 women). The study described the timing, location, cause, and treatment of falls. Fall prevalence was standardized to the 2006 Australian population. Data collection included self-reported prior fractures, medication, disease conditions, anthropometry, and biochemical tests. Binary logistic regression identified factors linked to fall-related injuries.

**Results:**

Fall rates by age group: 20–29 (24.4%), 30–39 (21.5%), 40–49 (18.7%), 50–59 (24.9%), 60–69 (25.0%), 70–79 (34.6%), 80+ (40.5%). The age-standardized prevalence of falls was 25.0% (95% CI: 23.4–26.7%). In adults ≥65 years, the age-standardized prevalence of falls was 32.4% (95% CI: 29.3–35.5%). Fallers were typically older, less mobile, had higher BMI and cholesterol levels, and were more likely to have musculoskeletal conditions, cancer, and polypharmacy. Men had a higher fall risk, but fewer fall-related fractures compared to women. Most falls occurred outdoors (62.1%), were due to extrinsic cause (58.5%), and were on the same level (78.5%). Factors associated with fall-related injuries included being female, elevated falls and falls outside the home. Nearly half (45.7%) of those injured sought medical attention, primarily from general practitioners (25.7%) and emergency departments (12.6%).

**Conclusion:**

Falls are common, occurring in approximately one quarter of adults in this study, with a greater prevalence among those ≥65 years. Women experienced fewer multiple falls than men. Many participants sought medical attention, primarily from general practitioners. This research identifies fall characteristics and circumstances, informing targeted prevention strategies to reduce occurrences and alleviate burdens on healthcare systems and individuals.

## Introduction

Every year, approximately 37 million falls occur globally, requiring medical attention, and resulting in over 17 million years lived with disability (1). Fatal falls, estimated at 684,000 annually, rank as the second leading cause of unintentional injury deaths globally, following traffic accidents (2). In Australia, falls and fall-related injuries are a growing concern (3), with one in three people aged ≥65 years experiencing a fall each year (4). Falls among older individuals incur a significant financial burden on the Australian healthcare system, costing approximately $2.3 billion annually (5). Fall-related injuries are a primary cause of trauma emergency admissions (3) and a major reason for hospitalizations and outpatient care by general practitioners (6).

The burden of falls varies across populations, with higher risks observed among aged care facility residents and hospital inpatients (4). Frailty, common among older patients in hospitals (7) and residential aged care (8), increases the risk of adverse outcomes such as falls (9). Falls are associated with chronic comorbid conditions (10, 11), including cardiometabolic, cardiovascular (12), cancer (13) and musculoskeletal conditions (14). Mental disorders, psychotropic medications (15), and excessive daytime sleepiness (16) are linked to fall risk but interestingly certain medications like statins, used in the treatment of high cholesterol levels can lower the risk of fall-related fractures (17).

Prevalence studies have been conducted in Asia, America, and Europe (18) but few have focused on Australians living in the community (3) and community care recipients (4). In Australia, fall prevention strategies include multi-component exercise programs, home safety modifications, Tai Chi programs, individualized risk assessments, education and training, community-based initiatives, and regular medication reviews to reduce fall risk (5). Preventing and managing falls in aging populations is crucial, as many older adults fall-related fatalities are rising but preventable (1). Australia plans to launch a national falls prevention strategy by 2025, aiming to reduce falls by 30% (5). A key step in developing this strategy is understanding the prevalence and characteristics of falls. Therefore, this study aimed to report the characteristics, circumstances, and treatment for falls and fall-related injuries in a population-based sample of Australian men and women.

## Methods

### Study design and population

Participants were from the Geelong Osteoporosis Study (GOS) (19), a cohort study involving residents of the Barwon Statistical Division in south-eastern Australia. The study recruited participants using a random sampling procedure from the Australian electoral roll, ensuring an even distribution of participants across the adult age range. Participants were deemed eligible for the study if they had resided in the region for at least 6 months and could provide informed consent. A cohort of 1,494 women aged 20–94 years was recruited from 1993 to 1997 (77% response), with subsequent follow-up measurements at multiple time intervals. Baseline visits for men occurred approximately a decade later, between 2001 and 2006, with 1,540 participants aged 20–92 years (67% response), followed by subsequent follow-up measurements at 5- and 15-year intervals (19). The data for this analysis were cross-sectional, drawn from the baseline visit for men (2001–2006) and the 10-year follow-up for women (2004–2008).

### Study variables

#### Fall variable

Falls data were collected via a self-reported questionnaire. A fall was defined as “when you suddenly find yourself on the ground without intending to get there, after you were in the lying, sitting or standing position” (19). Participants were asked whether they had experienced a fall during the past 12 months. Multiple falls were defined as having two or more falls over the previous 12 months. For participants who reported falls, additional questions were asked, including when it happened (month), location of the fall, description/cause of the fall, whether the fall was from a greater than standing height, any injuries sustained, and the type of treatment received (e.g., general practitioner (GP), emergency department, home treatment, physiotherapy) (Supplementary Table 1).

#### Location of falls

The variable “location of falls” was described in three different ways as follows (Supplementary Table 1):

- Inside home/Outside Home:

This category encompasses falls that occurred within the participant’s home. “Inside home” included falling out of bed, on stairs, in the bathroom, kitchen, etc. “Outside home” included falls that took place outside the participant’s home, such as in the backyard, garden, etc.

- Indoor/Outdoor:

“Indoor Falls” were classified as falls occurring within a building or under cover at work, including locations such as a garage, warehouse, factory, shopping center, hotel, nightclub, gymnasium, church, or inside the participant’s home. “Outdoor Falls” included falls occurring outside, such as a paddock, street, construction site, tennis court, netball court, golf course, cricket ground, football field, park on farmland, or outside the participant’s home.

- At Home/Other Places:

“At Home Falls” included all falls that occurred at home, encompassing both inside and outside falls within the participant’s home. “Other Places Falls” were falls that occurred in locations other than the participant’s home.

#### Cause of falls

The cause of falls was divided into intrinsic and extrinsic factors. Intrinsic factors are related to the individual, including fainting, blacking out, passing out, loss of leg strength, vertigo, dizziness, pinched nerve on legs, and similar factors. Extrinsic factors are from outside factors, such as slipping, tripping, being knocked down, or other environmental factors (Supplementary Table 1).

#### Fall-related injuries

Fall-related injuries were classified into two groups: no injury and injury (minor and major). Minor injuries included scratches, bruises, and superficial wounds requiring minimal medical attention, while major injuries encompassed sprains, serious head injuries, joint deformities or dislocations, contusions, cuts, unconsciousness, and fractures (20).

### Other variables

Weight was measured using electronic scales with an accuracy of 0.1 kg, while height was measured using a Harpenden stadiometer with an accuracy of 0.001 m. Body mass index (BMI) was determined by dividing the weight in kilograms by the square of the height in meters. Participants provided self-reported data on their mobility status, with mobility levels ranging from ‘very active’ to ‘unable to walk’ (19). Participants were also asked if they used a mobility assistive device such as a walker or cane. Medication use was assessed using a self-reported questionnaire. Polypharmacy was defined as the use of five or more medications as this cut-point has previously been associated with fall risk (21). Blood samples were obtained following an overnight fasting period and standard laboratory techniques were used to determine levels of fasting plasma glucose and cholesterol.

Comorbidities were categorized into the following groups: (i) pulmonary, (ii) musculoskeletal, (iii) cancer, and (iv) cardiometabolic conditions. Pulmonary diseases encompass self-reported asthma, emphysema, and chronic bronchitis. Musculoskeletal conditions were based on self-reported muscle weakness, osteoarthritis, and rheumatoid arthritis, as well as low femoral neck bone mineral density (BMD) and prior low trauma fractures (excluding fractures of the fingers, toes, skull, and face). Low femoral neck BMD (Lunar DPX-L; Lunar; Madison, WI, USA) and GE-Prodigy (Prodigy; GE Lunar, Madison, WI, USA) was defined as a T-score < −2.5, with cutoffs of 0.747 g/cm^2^ for men (22) and 0.701 g/cm^2^ for women (23). Cancer data were obtained by linkage with the Victorian Cancer Registry dating back to 1986. Cardiometabolic conditions included self-reported stroke or arrhythmias, hypertension (systolic blood pressure ≥ 140 mmHg and/or diastolic blood pressure ≥ 90 mmHg, measured in a seated position using an automated device), diabetes (self-report of diabetes/antihyperglycemic medication and/or fasting plasma glucose ≥7.0 mmol/L), obesity (BMI > 30 kg/m^2^), and hypercholesterolemia (total cholesterol ≥5.2 mmol/L) (24).

### Statistical analysis

The data were analyzed using Stata version 18 (Stata Corp. 2023. Stata Statistical Software: Release 18. College Station, TX: Stata Corp LLC). Descriptive statistics were employed to analyze participant characteristics, with analyses stratified by sex and age groups. Normally distributed continuous variables were summarized using mean and standard deviation (SD), while non-normally distributed continuous variables were summarized using median and interquartile range (IQR). Categorical variables were reported using frequencies and percentages. Two sample t-tests, the Mann–Whitney U test, and a Chi-squared test were employed to explore inter-group differences. The prevalence of self-reported falls over the previous 12 months was age standardized to the 2006 Australian population.

Factors associated with fall-related injuries were identified using a binary logistic regression model. A bivariate analysis was performed first between one independent variable and the outcome variable. Multivariable analysis was then applied to variables associated with the outcome variable at a *p*-value *<* 0.25 to control for potential confounding variables. Variables with a *p*-value >0.25 at the bivariate level, yet considered clinically significant, were incorporated into the multivariable analysis. Interaction terms were examined in the final models, and no significant interaction terms were identified. A two-sided test was performed, and the level of significance was declared at a *p*-value *<* 0.05.

## Results

Six men and 20 women were excluded as they did not recall whether they had a fall over the last 12 months, therefore, a total of 2,631 participants (1,533 men and 1,098 women) were included in the study. The median (IQR) age of the study participants was 54.0 (37.6–71.0) years, ranging from 20 to 97 years. Fallers were slightly older than non-fallers. The median (IQR) total cholesterol for fallers was 5.4 mmol/L (4.9–5.9), compared to 5.1 mmol/L (4.4–5.8) for non-fallers (*p* < 0.001). Fallers exhibited lower mobility, higher BMI, and cholesterol levels, and were more likely to have musculoskeletal conditions, cancer, and polypharmacy compared to non-fallers (*p* < 0.001; Table 1).

**Table 1:** Characteristics of the participants based on fall status.

Variables	All (*n* = 2,631)	Fallers (*n* = 708)	Non-fallers (*n* = 1,923)	*p*-value
Age (yr)	54.0 (37.6–71.0)	55.2 (39.1–83.0)	53.5 (37.4–67.8)	<0.001
Sex (Male)	1,533 (58.3)	422 (59.6)	1,111 (57.8)	0.385
Weight (kg)	78.4 ± 15.9	78.1 ± 16.7	78.6 ± 15.6	0.002
Height (m)	1.69 ± 0.1	1.68 ± 0.1	1.70 ± 0.1	0.001
BMI ^a^ (kg/m^2^)	27.2 ± 5.3	26.5 ± 5.7	27.5 ± 5.1	<0.001
Cholesterol-LDL ^b^ (mmol/L)	3.0 (2.5–3.6)	3.2 (2.8–3.8)	3.0 (2.4–3.6)	<0.001
Cholesterol-HDL ^c^ (mmol/L)	1.4 (1.2–1.6)	1.4 (1.3–1.7)	1.3 (1.1–1.6)	<0.001
Total cholesterol (mmol/L)	5.1 (4.5–5.8)	5.4 (4.9–5.9)	5.1 (4.4–5.8)	<0.001
Mobility				<0.001
Very active	584 (22.2)	217 (30.6)	367 (19.1)	
Active	1,420 (54)	321 (45.3)	1,099 (57.2)	
Sedentary	511 (19.4)	127 (17.9)	384 (19.9)	
Limited	96 (3.6)	34 (4.8)	62 (3.2)	
Inactive^#^	16 (0.6)	7 (1.0)	9 (0.5)	
Mobility device use	163 (6.2)	46 (6.5)	117 (6.1)	0.696
Polypharmacy ^d^	468 (17.8)	83 (11.7)	385 (20.0)	<0.001
Comorbidities				
Pulmonary ^e^	500 (19.0)	136 (19.2)	364 (18.9)	0.871
Musculoskeletal ^f^	744 (28.3)	237 (33.5)	507 (26.4)	<0.001
Cancer	539 (20.5)	109 (15.4)	430 (22.4)	<0.001
Cardio-metabolic ^g^	1,005 (38.2)	253 (35.7)	752 (39.1)	0.114
Cardiovascular	645 (24.5)	161 (22.7)	484 (25.2)	0.199

Data are presented as mean (±SD), median (IQR) or *n* (%).

a BMI, Body mass index.

b LDL, Low density lipoprotein.

c HDL, High density lipoprotein.

d Polypharmacy (defined as ≥ 5 medicines).

e Pulmonary diseases encompassed asthma, emphysema, and chronic bronchitis.

f Musculoskeletal conditions included muscle weakness, osteoarthritis, rheumatoid arthritis, low femoral neck bone mineral density (BMD) and prior low trauma fractures (excluding fractures of the fingers, toes, skull, and face).

g Cardiometabolic conditions included hypertension, stroke, arrhythmias, diabetes (self-report of diabetes/antihyperglycemic medication or fasting plasma glucose ≥ 7.0 mmol/L), obesity (BMI > 30 kg/m^2^), and hypercholesterolemia (TC ≥ 5.2 mmol/L). n, frequency; SD, standard deviation; IQR, interquartile range.

# Inactive includes inactive, chair or bedridden and bedfast.

### Prevalence of falls

**Figure 1:**
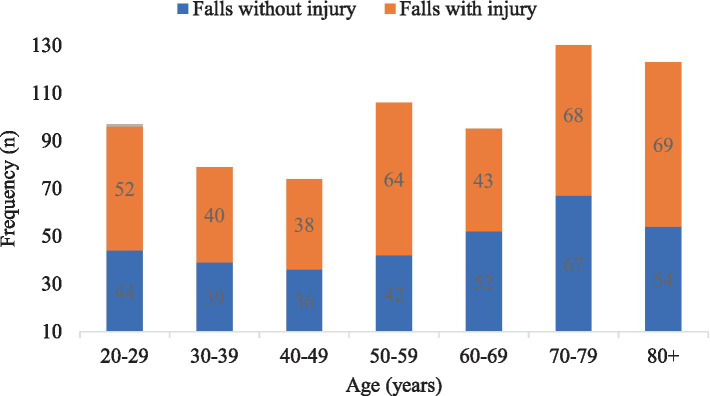
The frequency of falls with and without injury across different age groups among participants in this study.

The 12-month prevalence of self-reported falls among all participants was 26.9% (95% CI: 25.2, 28.7%). After age-standardization, the prevalence estimate was 25.0% (95% CI: 23.4, 26.7%). Among older adults aged ≥65 years (*n* = 871; 33.1%), the 12-month prevalence of self-reported falls was 34.3% (95% CI: 31.1, 37.6%). After age-standardization, the prevalence was 32.4% (95% CI: 29.3, 35.5%). There were 957 fall events over the previous 12 months, reported by 708 participants. The prevalence of fallers varied across age groups (*p* < 0.001; Figure 1), but there was no significant difference between sex groups (27.5% for men, 26.1% for women, *p* = 0.399).

The age-standardized prevalence of falls increased with age, with estimated prevalences of 23.1, 31.6, and 46.8% among age groups of 20–64 years, 65–84 years, and 85+ years, respectively. The prevalence of sport-related falls was 7.3% (Table 2). Among those ≥85 years old, 8.3% experienced multiple falls, compared to 6.9% who had a single fall (Table 3). The age-standardized prevalence of multiple falls over the past 12 months among individuals aged 20–97 years was 5.9% (95% CI: 5.0, 6.8%). Multiple falls were more common among men (4.3%) compared to women (2.1%) (*p* = 0.033), but did not differ across age groups (*p* = 0.716). Sixty-one participants (2.3%) experienced three fall events, and 20 participants (0.8%) experienced four fall events (Supplementary Table 2).

**Table 2:** Description of the participant characteristics by falls and fall-related injuries.

Variables	Categories	Fallers (*n* = 708)	No injury (*n* = 190)	Injury (*n* = 374)^#^
		*n* (%)	*n* (%)	*n* (%)
Age groups (yr)	20–64	409 (57.8)	118 (62.1)	217 (58)
	65–84	248 (35.0)	58 (30.5)	127 (34)
	85+	51 (7.2)	14 (7.4)	20 (8.0)
Sex	Male	422 (59.6)	129 (67.9)	187 (50.0)
Location of falls	Indoor	241 (34.0)	67 (35.3)	132 (35.3)
	Outdoor	440 (62.2)	119 (62.6)	241 (64.4)
	Inside home	169 (23.9)	51 (26.8)	88 (23.5)
	Outside home	202 (28.5)	59 (31.1)	107 (28.6)
	At home	371 (52.4)	110 (57.9)	195 (52.1)
	Other places	336 (43.8)	76 (40.0)	178 (47.6)
Cause of falls	Intrinsic	258 (36.4)	63 (33.2)	132 (36.6)
	Extrinsic	414 (58.5)	120 (63.2)	227 (60.7)
Falls from greater height	Yes	125 (17.7)	29 (15.3)	74 (20.8)
Sport-related falls	Yes	52 (7.4)	14 (7.4)	28 (7.5)
Treatment	Yes	180 (25.4)	9 (4.7)	171 (45.7)

n, frequency.

# Missing data for unknown fall-related injury *n* = 144.

**Table 3:** Description of the characteristics of falls for participants with a single or multiple (≥2) falls over the past 12 months.

Variables	Categories	Multiple (≥2) falls (*n* = 168)	Single fall (*n* = 540)	*p*-value
		*n* (%)	*n* (%)	
Age groups (yr)	20–64	94 (56.0)	315 (58.3)	0.761
	65–84	60 (35.7)	188 (34.8)	
	85+	14 (8.3)	37 (6.9)	
Sex	Male	112 (66.7)	310 (57.4)	0.033
Location of falls	Indoor	52 (31.0)	189 (35.0)	0.112
	Outdoor	99 (58.9)	341 (63.2)	
	Inside home	42 (25.0)	127 (23.5)	0.163
	Outside home	57 (33.9)	245 (45.4)	
	At home	99 (58.9)	272 (50.4)	0.082
	Other places	52 (41.0)	238 (44.1)	
Cause of falls	Intrinsic	68 (40.5)	190 (35.2)	0.282
	Extrinsic	94 (59.5)	320 (59.3)	
Falls from greater height	Yes	20 (11.9)	105 (19.4)	0.072
Sport-related falls	Yes	9 (5.4)	43 (8.0)	0.111
Injury	Yes	67 (39.9)	311 (57.6)	0.176
Treatment	Yes	28 (16.7)	152 (28.2)	0.742

### Cause of falls

One hundred twenty-five (17.7%) participants experienced falls from greater than standing height (elevated falls) (Table 2). Intrinsic factors were present in 40.5% of the multiple falls group and 35.2% of the single falls group (Table 3). Extrinsic cause of falls was the most common cause, reported by 58.5% of fallers. In comparison to intrinsic cause, extrinsic cause was more prevalent among both men (56.2% vs. 37.7%) and women (61.9% vs. 34.6%) (Table 4). Among 374 fall-related injuries, 227 (60.7%) were attributed to extrinsic causes of falls.

**Table 4:** Description of participant characteristics of falls by sex and age groups.

Variables	Categories	Sex		Age groups		
		Male (*n* = 422)	Female (*n* = 286)	20–64 yr (*n* = 409)	65–84 yr (*n* = 248)	85+ yr (*n* = 51)
		*n* (%)	*n* (%)	*n* (%)	*n* (%)	*n* (%)
Sex	Male	–	–	224 (54.8)	161 (64.9)	37 (72.6)
Location of falls	Indoor	127 (30.1)	114 (39.9)	134 (32.8)	85 (34.3)	22 (43.1)
	Outdoor	273 (64.7)	167 (58.4)	257 (62.8)	156 (62.9)	27 (52.9)
	Inside home	84 (19.9)	85 (29.7)	85 (20.8)	67 (27.0)	17 (33.3)
	Outside home	126 (29.9)	76 (26.6)	87 (21.3)	101 (40.7)	14 (27.5)
	At home	210 (49.8)	161 (56.3)	172 (42.1)	168 (67.7)	31 (60.8)
	Other places	190 (45.0)	120 (42.0)	219 (53.6)	73 (29.4)	18 (35.3)
Cause of falls	Intrinsic	159 (37.7)	99 (34.6)	149 (36.4)	90 (36.3)	19 (37.3)
	Extrinsic	237 (56.2)	177 (61.9)	259 (63.3)	147 (59.3)	28 (54.9)
Falls from greater height	Yes	85 (20.1)	40 (14.0)	87 (21.3)	32 (12.9)	6 (11.8)
Sport-related falls	Yes	35 (4.9)	17 (2.4)	49 (6.9)	2 (0.3)	1 (0.14)
Fracture	Yes	17 (2.4)	30 (4.2)	27 (3.8)	18 (2.5)	2 (0.3)
Treatment	Yes	95 (13.4)	85 (12.0)	106 (15.0)	58 (8.2)	16 (2.3)

*n, frequency.*

### Location of falls

A higher proportion of falls occurred outside the home compared to inside the home (28.5% vs. 23.9%) (Table 2). Falls inside the home occurred in 33.9% of the multiple falls group and 23.5% of the single falls group (Table 3). Among men, falls outside the home were more prevalent than inside (29.9% vs. 19.9%), whereas for women, the trend was reversed (26.6% vs. 29.7%) (Table 4). Outside-the-home falls were common among individuals aged 20–64 years (21.3% vs. 20.8%) and 65–84 years (40.7% vs. 27%), but not among those aged 85+ years (27.5% vs. 33.3%) (Table 4).

**Figure 2:**
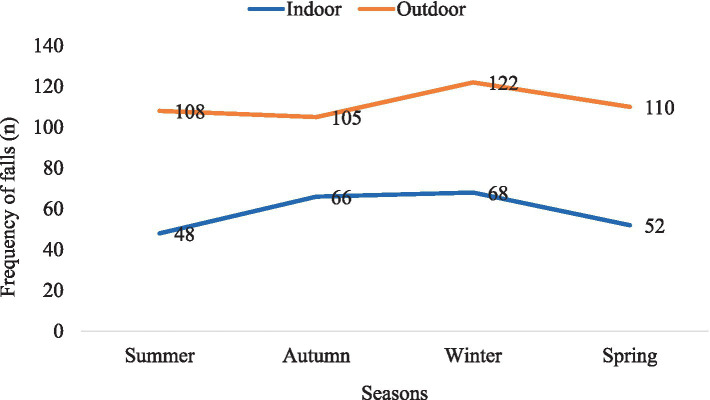
The frequency of falls by location of falls across different seasons among participants in this study.

Many individuals who experienced falls reported them occurring outdoors (62.6%, Table 2). Outdoor falls were more frequent among both men (64.7% vs. 30.1%) and women (58.4% vs. 39.9%) compared to indoor falls (Table 4). Across all age groups, outdoor falls were more common than indoor falls (Table 4). Additionally, outdoor falls were more prevalent than indoor falls across all seasons, with winter showing a particularly higher occurrence (Figure 2).

Over 52.4% of individuals who experienced falls reported them occurring at home (Table 2). Both men and women reported falls at home more frequently compared to falls in other locations (Table 4). Falls at home were notably more prevalent among age groups 65–84 years (67.7% vs. 29.4%) and 85+ years (60.8% vs. 35.3%), but not among those aged 20–64.99 years (42.1% vs. 53.6%) (Table 4).

### Fall-related injury

The age-standardized prevalence of fall-related injuries for individuals aged 20–97 years was 13.2% (95% CI 11.9, 14.5%). Fall-related injury was higher among women (*p* < 0.001) but did not differ across age groups (Figure 1; *p* = 0.646). Among 708 fallers, 256 (36.2%) experienced minor injuries. Specifically, 24.2% had bruising, 7.4% suffered a sprain, 1.7% endured a strain, and 1% had a concussion. The fracture rate among the fallers was 6.6%. The fracture rate varied across sex groups, with 2.4% for men and 4.2% for women (*p* < 0.001), but not across age groups (*p* = 0.514). Factors associated with fall-related injuries included being female, elevated falls, and falls outside the home (Table 5).

**Table 5:** Factors associated with fall-related injury among participants in this study.

Variables	Categories	COR (95% CI)	*p*-value	AOR (95% CI)	*p*-value
Age groups (yr)	20–64	1		1	
	65–84	1.19 (0.81, 1.75)	0.372	1.49 (0.97, 2.31)	0.071
	85+	1.17 (0.60, 2.28)	0.656	1.78 (0.83, 3.84)	0.140
Sex	Male	1		1	
	Female	2.11 (1.46, 3.05)	<0.001	2.34 (1.44, 3.96)	0.001
Falls from greater height	No	1		1	
	Yes	1.39 (0.87, 2.22)	0.171	1.78 (1.03, 3.06)	0.038
Location of falls	At home	1		1	
	Other places	1.32 (0.93, 1.89)	0.195	1.49 (1.02, 2.19)	0.040
Cause of falls	Intrinsic	1		1	
	Extrinsic	0.87 (0.60, 1.26)	0.462	0.98 (0.65, 1.49)	0.938
Cardiometabolic conditions	No	1		1	
	Yes	0.60 (0.42, 0.87)	0.007	1.00 (0.61, 1.66)	0.985
Musculoskeletal conditions	No	1		1	
	Yes	1.41 (0.97, 2.05)	0.076	1.13 (0.76, 1.69)	0.537

AOR, adjusted odds ratio; CI, confidence interval; COR, crude odds ratio; 1, reference group.

### Treatment

**Figure 3:**
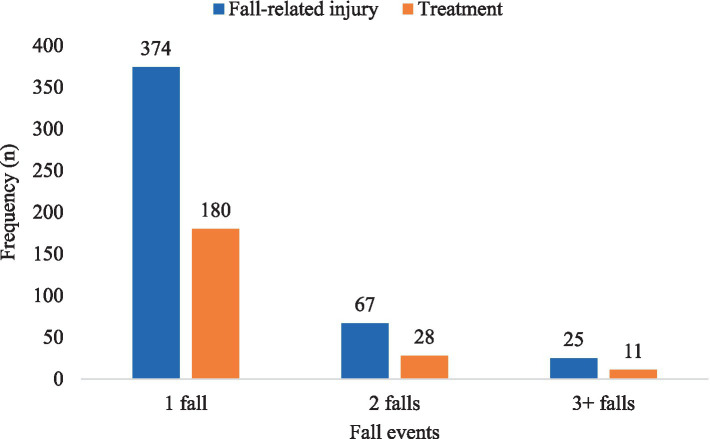
The frequency of fall-related injury and treatment sought across different fall events among participants in this study.

The overall proportion of participants who sought treatment after a fall (regardless of injury status) was 180/708 (25.4%). Of those who have sustained an injury, a greater proportion, 171/374 (45.7%) sought treatment. When considering all reported fall events (including multiple falls), less than half of the injured respondents sought treatment (Figure 3). Among those who sustained injuries, the majority were treated by a general practitioner (25.7%), followed by 19.5% who received other treatments (e.g., osteopathy, surgery, imaging tests), and 12.6% who were treated at the emergency department (Supplementary Table 2). Additionally, 16% sought multiple treatments simultaneously.

## Discussion

In this study, we report the characteristics, circumstances, and treatment for falls and fall-related injuries in a population-based sample of Australian men and women. We report an age-standardized prevalence of 25.0% for falls among individuals aged 20–97 years over the past 12 months. Furthermore, the prevalence of falls increased with age. Fallers tended to be older, less mobile, with higher BMI and cholesterol levels, and more likely to have musculoskeletal conditions, cancer, and polypharmacy.

Our study reports a 25.0% age-standardized prevalence of falls among individuals aged 20–97 years; below the prevalence of 30% reported among participants ≥18 years in a study from Japan (25). In our study, we report that the age-standardized prevalence of falls for older adults aged ≥65 years was 32.4%. This finding is consistent with two studies conducted in Malaysia (31.4 and 32.2%) (26, 27) and Turkey (32.1%) (28). However, the prevalence in our study was higher than 27.6, and 28.1% reported in Brazil (29, 30), and 20.65% in China (31). A systematic review and meta-analysis encompassing 37 studies involving community-dwelling residents, revealed a lower prevalence of falls of 27% (32). Similarly, a study from 17 European countries and Israel reported a prevalence of falls at 8.2%, with higher estimates observed in women and increasing with age (33). However, this finding was lower than the 39.7% higher prevalence of falls in Tehran (34), with a higher occurrence in women compared to men (34). Similarly, most falls in our study, as well as in Tehran (34), occurred at home. The differences in estimates between this study and others can be attributed to variations in sample size, the population under study, the age range of participants, and the duration of follow-up. In contrast to our study, most other studies utilized smaller sample sizes, focused on attendees of health clinics (27), targeted a younger age group starting from ≥50 years (26), and employed a 6-month follow-up period to assess the prevalence of falls (33).

In a study conducted in Saudi Arabia involving 280 older adults aged >60 years, the prevalence of falls within the previous year was 31.6%. Women exhibited a higher frequency of falls compared to men (34.5% versus 28.5%), with most falls occurring indoors (84.7%). Advanced age, polypharmacy, and environmental factors were identified as significant risk factors for falls (35). Similarly, a study by Galvan et al. (36), which included 350 older adults aged ≥80 years, reported a prevalence of falls of 46.9%. Extrinsic factors were identified as the most common cause of falls. These results are consistent with the findings of the present study, which identified fallers were more likely to have polypharmacy and a greater prevalence of extrinsic (environmental) factors among fallers compared to non-fallers (58.5% vs. 36.4%).

Outdoor falls were found to be more prevalent than indoor falls across various demographic categories including sex, age groups, and throughout all seasons. This observation is corroborated by a similar study conducted somewhere (37). In Victoria, Australia, falls are more common in winter due to wet and slippery conditions from rainfall, frost, or occasional snow at higher elevations. Older adults and individuals with mobility issues are more vulnerable to fall risks during winter due to reduced daylight hours. A significant proportion of outdoor falls have been linked to modifiable environmental factors, such as uneven surfaces and obstacles, typically encountered on footpaths, curbs, and streets within urban areas. Moreover, most falls were observed to have transpired outdoors rather than indoors (62.2% vs. 34.0%).

Interestingly, across all age groups, men exhibited a higher annualized rate of falls compared to women, yet they experienced fewer fall-related injuries. This finding contradicts previous studies where fall rates were notably higher among women than men (28, 29, 32). Similarly, a study from 17 European countries and Israel reported higher estimates observed in women and increasing with age (33). Contrary to our study, Sotoudeh et al. (34) observed a greater prevalence of falls in women compared to men. The difference could be attributed to the fact that, unlike the study by Sotoudeh et al. (34), which included a greater proportion of women, our study included more men. Additionally, the age difference may create a discrepancy in the prevalence of falls. Unlike our study, which includes a range of individuals aged 20–97 years, Sotoudeh et al. (34) included only individuals aged ≥65 years. Nevertheless, like our findings, they also observed that most falls in their study occurred at home.

The age-standardized prevalence of multiple falls over the past 12 months among individuals aged 20–97 years was 5.9%. This finding closely aligns with the 5.4% multiple falls reported by Uno et al. (25). However, it was notably lower than the 26.1% reported by Curran-Groome et al. (38), whose study focused on admitted patients in a trauma surgery department. The observed disparities can be attributed to the higher likelihood of falling in hospital and aged care settings compared to community dwellers (4).

Research has consistently demonstrated a relationship between BMI and falls, often characterized by a U-shaped pattern, indicating that both low and high BMI levels are associated with an increased risk of falls (39). In our research, individuals who experienced a fall had a higher mean BMI. Higher BMI might be linked to factors like reduced balance or mobility, potentially elevating the risk of falling. Unhealthy body habits, such as higher BMI, are consistently associated with a heightened fall risk, possibly due to the presence of underlying comorbidities (40, 41) or medications used for treatment (42), and in cases of polypharmacy (43).

Our research identified a connection between falls and musculoskeletal conditions, such as muscle wasting, osteoarthritis, rheumatoid arthritis, and low femoral bone mineral density. These conditions increase fall risk due to pain and poor balance (44). Afrin et al. (45) found that individuals with musculoskeletal disorders are at higher risk of falls and injuries.

Our research identified an association between cancer and falls. Cancer-induced cachexia, characterized by significant weight, fat, and muscle loss (46), contributes to physical decline and increases the likelihood of falls (13). Cachexia is a syndrome causing severe weight, fat, and muscle loss, often linked to chronic illnesses like cancer. Individuals with cancer are more prone to experiencing functional impairment, frailty, and dementia, which collectively contribute to the occurrence of falls compared to those without cancer (13).

The age-standardized prevalence of fall-related injuries in our study was 13.2% (95% CI 11.9, 14.5%). This finding is consistent with 14.48% (31) in China. Across various studies, the prevalence of fall-related injuries has shown considerable variability. This finding is higher than 8.7% in China (47) and 11% in Brazil (30). However, this finding is lower than 38.2% among aged care residents in Canada (48) and 49.1% of a 6-month prevalence in Serbia (49).

According to our research, factors such as sex, the level of the fall, and the location of the fall are all associated with the likelihood of sustaining an injury from a fall. Women had a higher likelihood of injuries related to falls than men. There was a 2.3-fold increase in injury risk among women who fell compared with men. This finding was supported by studies conducted elsewhere (50, 51). Furthermore, the risk of non-fatal fall-related injuries, particularly fractures, was found to be higher in women than in men (52). This could be attributed to disparities in fall-related injuries, such as fracture rates, potentially explained by the fact that women experience greater bone loss due to menopause (53). Additionally, women tend to experience more rapid deterioration in lower limb muscular strength as they age compared to men (11), further increasing their susceptibility to falls and fall-related injuries.

Fall-related injuries were found to be associated with the level of falls that occurred. Specifically, participants who fell from greater heights (elevated falls) had a 1.8-fold increase in the likelihood of developing a fall-related injury compared to those who experienced same-level falls. Studies conducted in various locations suggest that falls from elevated heights are more likely to cause fall-related injuries due to the increased impact forces upon landing (54, 55). Research, including that by Hsieh et al. (56) indicates that individuals experiencing elevated falls face a higher risk of severe injury and mortality compared to those with same-level falls. These findings emphasize the significance of considering the height of falls when assessing and treating fall-related injuries.

Our research identified an association between fall-related injuries and the location of falls. Specifically, individuals who fell in a location other than at home had a 1.5-fold higher likelihood of sustaining an injury compared to those who fell at home. This finding aligns with another study conducted elsewhere, which reported similar results (55). However, it is important to note that there are differing findings in the literature. For instance, Mekkodathil et al. (54) revealed that older adults who fell at home were more likely to sustain fall-related injuries. These discrepancies highlight the complexity of factors influencing fall-related injuries and underscore the need for further research to better understand these associations. Our findings indicate a difference in the location of falls across age groups. This is consistent with the study by Stevens et al. (52), which found that older people are more likely to experience falls at home compared to younger individuals.

The study discovered that nearly half (48.1%) of the individuals who sustained fall-related injuries sought medical attention. Among those seeking care, the majority visited their general practitioner, with subsequent visits to a hospital emergency department. Watson et al. (57) highlighted that hospital treatment associated with fall-related injuries constituted 58% of all treatments, with medical treatments from general practitioners and specialists making up 6% of hospital treatments, and emergency rooms accounting for 4%. Additionally, a study involving residents from the state of New South Wales in Australia reported that 20% presented to a hospital due to a fall, indicating the substantial impact of fall-related injuries on healthcare utilization (3).

According to our research, 6.2% of individuals who sustained injuries sought out physiotherapy as a therapeutic option. Physiotherapy, as highlighted by Sherrington et al. (58), is not only used for rehabilitation but also for fall prevention and reducing the risk of injuries resulting from falls. Interestingly, our study found that 4.6% of injured individuals were self-treated at home. Watson et al. (57) revealed that most of the treatment (69%) occurs outside the hospital setting, indicating the importance of considering various treatment options and settings in managing fall-related injuries. Injuries stemming from falls are associated with significant treatment costs. Fall prevention programs should target adults aged ≥65 years, incorporating strategies like exercise, home modifications, and individualized risk assessments. Routine fall risk assessments should be included in check-ups, and public awareness should focus on modifiable risk factors. Policymakers need to support community-based fall prevention efforts to meet the needs of the aging population.

### Strength and limitations

The main strength of this study is its large sample size, with over 2,600 participants, providing adequate statistical power. The participants were also randomly selected from the electoral roll and are representative of the broader Australian population. While self-reported bias due to relying on participants to recall and report falls (59) may lead to underestimation, particularly for minor falls or those not resulting in injury, our method of identifying falls has previously been shown to be reliable (60). The study used similar methods to recruit men and women from the same population at around the same time, making cohort effects unlikely. The cross-sectional nature of the study prevents the establishment of causality.

## Conclusion

Falls are common, reported by about one-quarter of the study participants, with higher prevalence among older individuals. Men had a higher risk of falling but experienced fewer fall-related fractures compared to women. One-fourth of participants sought medical care from general practitioners, regardless of injury. This research identifies fall characteristics and circumstances, informing targeted prevention strategies to reduce occurrences and alleviate burdens on healthcare systems and individuals.

## Data Availability

The raw data supporting the conclusions of this article will be made available by the authors, upon reasonable request.
