# Supplementary material for: Falls and fall-related injuries: prevalence, characteristics, and treatment among participants of the Geelong Osteoporosis Study
Source: Front Public Health. 2024 Oct 18;12:1454117. doi: 10.3389/fpubh.2024.1454117 (PMC11527698; doi:10.3389/fpubh.2024.1454117)
Supplement: Supplementary file 1 [file Table_1.docx]

**Supplementary file**

Supplementary table 1: Measurement and operational definitions of variables in the study.

| S. N | Variable | Explanation |
| --- | --- | --- |
| 1 | Age | The age of the respondents is categorised and coded as follows:  Category 1: Age range from 20 to 64 years, coded as "1".  Category 2: Age range from 65 to 84 years, coded as "2".  Category 3: Age range from 85+ years, coded as "3". |
| 2 | Falls | Categorised as:  "Non-faller": Coded as "0" if the participant responded "no" to the question "have you had a fall during the past year?"  "Faller": Coded as "1" if the participant responded "yes" to the question "have you had a fall during the past year?" |
| 3 | Location of falls | The analysis of the location of falls was conducted in three different ways:   1. Inside/Outside Home:   Inside Falls: This category encompasses falls that occurred within the participant's home. Examples include falling out of bed, on home stairs, in the bathroom, kitchen, etc.  Outside Falls: These are falls that took place outside the participant's home, such as in the backyard, garden, etc.   1. Indoor/Outdoor Location:   Indoor Falls: Falls occurring within a building or under cover at work, including locations such as a garage, warehouse, factory, shopping centre, hotel, nightclub, gymnasium, church, or inside the participant's home.  Outdoor Falls: Falls happening outside, such as a paddock, street, construction site, tennis court, netball court, golf course, cricket ground, football field, park on farmland, or outside the participant's home.   1. At Home/Other Places:   At Home Falls: This category includes all falls that occurred at home, encompassing both inside and outside falls within the participant's home.  Other Places Falls: These are falls that happened in locations other than the participant's home. |
| 4 | Causes of falls | Categorised as:  "Intrinsic causes": Coded as "0" if the causes are related to the individual, including fainting, blacking out, passing out, loss of leg strength, vertigo, dizziness, pinched nerve on legs, and similar factors.  "Extrinsic causes": Coded as "1" if the causes are from outside factors, such as slipping, tripping, being knocked down, or other environmental factors. |
| 5 | Injury | Categorised as:  "No injury": Coded as "0" if respondents did not sustain any injury.  "Injury": Coded as "1" if respondents had a fall-related injury such as a fracture, sprain, strain, or any other injury resulting from a fall. |
| 6 | Treatment | Categorised as:  "Not treated": Coded as "0" if no treatment was sought for a fall-related injury.  "Treated": Coded as "1" if treatment was sought, including GP, emergency, home treatment, physiotherapy, and various other treatments such as osteopathy, chiropractic care, surgery (e.g., hip replacement, laser surgery), imaging tests (e.g., magnetic resonance imaging, computed tomography, ultrasound), arthroscopy, blood tests, pain medication, and antibiotics. |

Supplementary table 2: Description of the characteristics of falls across fall events among participants in this study.

| Variables | Categories | 1 fall  n (%) | 2 falls  n (%) | 3+ falls  n (%) |
| --- | --- | --- | --- | --- |
| Falls events | | 708 (26.9) | 168 (6.4) | 81 (3.1) |
| Location of falls | Inside home | 169 (23.9) | 45 (26.8) | 22 (27.2) |
|  | Outside home | 202 (28.5) | 41 (24.4) | 17 (21.0) |
|  | Indoor | 241 (34.0) | 57 (33.9) | 30 (37.0) |
|  | Outdoor | 440 (62.1) | 107 (63.7) | 50 (61.7) |
|  | At home | 371 (52.4) | 86 (51.2) | 39 (48.1) |
|  | Other places | 336 (47.5) | 78 (46.4) | 41 (50.6) |
|  | Unknown | 1 (0.1) | 4 (2.4) | 1 (1.2) |
| Causes of falls | Intrinsic | 258 (36.4) | 65 (38.7) | 38 (46.9) |
|  | Extrinsic | 414 (58.5) | 94 (56.0) | 35 (43.2) |
|  | Unknown | 36 (5.1) | 9 (5.3) | 8 (9.9) |
| Falls from greater height | Yes | 125 (17.7) | 22 (13.1) | 9 (11.1) |
|  | No | 556 (78.5) | 142 (84.5) | 67 (82.7) |
|  | Unknown | 27 (3.9) | 4 (2.4) | 5 (6.2) |
| Fall-related injury | Yes | 374 (52.8) | 67 (39.9) | 25 (30.9) |
|  | No | 190 (26.8) | 45 (26.8) | 25 (30.9) |
|  | Unknown | 144 (20.3) | 56 (33.3) | 31 (38.3) |
| Treatment | Yes | 180 (25.4) | 28 (16.7) | 11 (13.6) |
|  | No | 194 (27.4) | 58 (34.5) | 23 (28.4) |
|  | Unknown | 334 (47.2) | 82 (48.8) | 47 (58.0) |
| Treatment types (more than one reported by respondents) (n = 374) | GP | 96 (25.7) | 12 (17.9) | 7 (28.0) |
|  | Emergency department | 47 (12.6) | 9 (13.4) | 4 (16.0) |
|  | Home treatment | 17 (4.6) | 3 (4.5) | 1 (0.1) |
|  | Physiotherapy | 23 (6.2) | 4 (6.0) | 0 |
|  | Others | 73 (19.5) | 4 (6.0) | 0 |

n = frequency; GP = general practitioner
